# Modified quadruple therapy versus bismuth-containing quadruple therapy in first-line treatment of *Helicobacter pylori* infection in Korea; rationale and design of an open-label, multicenter, randomized controlled trial

**DOI:** 10.1097/MD.0000000000013245

**Published:** 2018-11-16

**Authors:** Hyun Lim, Chang Seok Bang, Woon Geon Shin, Jae Ho Choi, Jae Seung Soh, Ho Suk Kang, Young Joo Yang, Ji Taek Hong, Suk Pyo Shin, Ki Tae Suk, Jae Jun Lee, Gwang Ho Baik, Dong Joon Kim

**Affiliations:** a Department of Internal Medicine; b Institute of New Frontier Research; c Department of Anesthesiology and Pain Medicine, Hallym University College of Medicine, Chuncheon, Korea.

**Keywords:** bacterial, bismuth, disease eradication, drug resistance, *Helicobacter pylori*

## Abstract

**Background:**

Clarithromycin-containing triple regimen for eradication of *Helicobacter pylori* is no longer acceptable in Korea due to high clarithromycin resistance. Concomitant therapy or bismuth-containing quadruple therapy is recommended as an alternative regimen. A recent study in Korea has shown that modified quadruple therapy has comparable efficacy and safety to concomitant therapy as a first-line regimen. However, there has been no comparative study of modified quadruple therapy with bismuth-containing quadruple therapy. The aim of this study is to compare the efficacy and safety of modified quadruple therapy with those of bismuth-containing quadruple therapy as a first-line regimen and to present the phenotypic and genotypic antibiotic resistance profile of *H pylori*.

**Methods:**

This study is an open-label, multicenter, randomized controlled trial. We are recruiting subjects endoscopically diagnosed with *H pylori* infection from 2 hospitals in Korea. Subjects will be randomly allocated either to modified quadruple therapy (proton-pump inhibitor bid, amoxicillin 1 g bid, metronidazole 500 mg tid, bismuth subcitrate 300 mg qid daily) or bismuth-containing quadruple therapy (proton-pump inhibitor bid, tetracycline 500 mg qid, metronidazole 500 mg tid, bismuth subcitrate 300 mg qid daily) for 14 days. The rate of eradication success and adverse events will be checked at least 4 weeks after the treatment. Antibiotic resistance will be established using both a bacterial culture with agar dilutions and DNA sequencing of the clarithromycin resistance point mutations in the 23S rRNA gene of *H pylori.*

**Conclusion:**

The results of this study will provide solid evidence for determining the optimal treatment regimen for first-line *H pylori* eradication in Korea.

## 1 Introduction

*Helicobacter pylori* is the most important pathogen in the development of gastric cancer.^[1,2]^ Infection with *H pylori* causes chronic atrophic gastritis, intestinal metaplasia, low- and high-grade dysplasia, and gastric cancer in sequence.^[3]^ Previous studies have shown the primary and secondary preventative effects of *H pylori* eradication in the development of gastric cancer,^[4–7]^ and eliminating this pathogen is considered the most promising strategy for the prevention of this disease.^[8]^

South Korea has the highest incidence of gastric cancer worldwide, and *H pylori* infection is still prevalent.^[9–11]^ Clarithromycin-containing triple therapy is the primary regimen approved and reimbursed by the Korean National Health Insurance Service.^[12]^ However, due to high clarithromycin resistance in Korea, the current eradication rate of clarithromycin-containing triple therapy has fallen below 80%, which is not acceptable as a first-line eradication regimen.^[9,13–15]^ Concomitant therapy or bismuth-containing quadruple therapy is recommended as an alternative regimen.^[8,9,12]^ However, concomitant therapy is not recommended in regions with high metronidazole resistance, and the resistance rate of metronidazole has been reported to be as high as 66% in Korea.^[9,16]^ Moreover, dual clarithromycin and metronidazole resistance undermines the efficacy of concomitant therapy, and there is no definite 2nd-line or 3rd-line rescue regimen for patients who do not respond to the above therapies.^[17,18]^ Therefore, finding an optimal regimen for 1st-line eradication is warranted in Korea.

A recent study in Korea has shown that modified quadruple therapy (proton-pump inhibitor (PPI), amoxicillin, metronidazole, bismuth subcitrate) has rates of eradication success and adverse events comparable to those of concomitant therapy.^[19]^ However, this study did not explore the antibiotic resistance profile of *H pylori*, and there has been no comparative study of modified quadruple therapy with bismuth-containing quadruple therapy. The aim of this study is to compare the efficacy and safety of modified quadruple therapy and bismuth-containing quadruple therapy and to present the phenotypic and genotypic antibiotic resistance profile of *H pylori*.

## 2 Methods

### 2.1 Overview of the trial design

Within this open-label, multicenter, randomized controlled, parallel-group trial, subjects with *H pylori* infection will be randomly assigned either to modified quadruple therapy (PPI bid, amoxicillin 1 g bid, metronidazole 500 mg tid, bismuth subcitrate 300 mg qid daily) or bismuth-containing quadruple therapy (PPI bid, tetracycline 500 mg qid, metronidazole 500 mg tid, bismuth subcitrate 300 mg qid daily) for 14 days. The eradication success rate, adverse events related to the medications and compliance will be investigated and compared between the 2 therapies. Phenotypic and genotypic antibiotic resistance testing will be performed during the trial, and these data will be used in the interpretation of the results.

Participants will be recruited from 2 Hallym University-affiliated Hospitals (Hallym University Chuncheon Sacred Heart Hospital in Chuncheon city, Hallym University Sacred Heart Hospital in Anyang city), and the recruitment period is from August 2018 to July 2019. Data will be collected from the subjects following their 1st hospital visit post-regimen (2 weeks after enrollment, after ending their eradication regimen) to check for adverse events related to the medications and for drug compliance and following their 2nd visit post-regimen (6 weeks after enrollment) to determine eradication success and to check for potential adverse events. The visit window is 1 week.

### 2.2 Study subjects

Subjects who undergo upper gastrointestinal endoscopy within 3 months and are proven to have *H pylori* infection either by the rapid urease test, the ^13^C-urea breath test (UBT), or a histological examination will be included in this trial. Exclusion criteria are as follows:

1. participants who have a history of *H pylori* eradication;
2. participants who have undergone a stomach resection;
3. participants who have a history of allergy or adverse events related to the eradication medications;
4. participants who have taken a PPI within 2 weeks or a histamine 2 receptor antagonist (H2RA) within 1 week;
5. participants who have taken aspirin (except low-dose aspirin for primary prophylaxis of cardiovascular disease), intravenous or oral nonsteroidal anti-inflammatory drugs, anticholinergics, prostaglandin analogs, pro-motility drugs, or sucralfate within a week or participants who need a continuous administration of those drugs;
6. participants who have taken antibiotics within 4 weeks;
7. pregnant or breast-feeding participants or participants who do not wish to avoid pregnancy during the clinical trial;
8. participants who are currently taking lovastatin, simvastatin, atorvastatin, indinavir, ritonavir, cyclosporin, terfenadine, pimozide, astemizole, human immunodeficiency virus protease inhibitors (atazanavir, nelfinavir), ergotamine, dihydroergotamine, mizolastine, bepridil, or ticagrelor;
9. participants who have infectious mononucleosis, a central nervous system infection, a hematological disease, galactose intolerance, Lapp lactase deficiency, glucose-galactose malabsorption, or Torsades de pointes; and
10. participants who are less than 18 years of age.

### 2.3 Outcome measurement

The *H pylori* infection is diagnosed by 1 or more of the following methods: the rapid urease test, the UBT or a histological examination. Two specimens from the gastric corpus and antrum will be taken during an endoscopy for the rapid urease test (Pronto Dry New; Medical Instruments Corp., Herford, Germany) or a histological assessment using Giemsa staining. A UBT (UBiT-IR 300; Otsuka Pharmaceutical Co., Ltd, Tokyo, Japan) measuring exhalation of ^13^CO_2_ before and 30 minutes after ingestion of 75 mg of ^13^C-marked urea will be performed. An initial breath sample will be obtained after at least an 8-hour fasting period (overnight fasting). The UBT after eradication of *H pylori* will be performed at least 4 weeks after the eradication therapy has ended. Delta over baseline >2.5% will be considered positive. Subjects who take medications that may affect the outcome of the UBT (e.g., PPI, H2RA, antibiotics) after the end of the eradication regimen will be tested at least 4 weeks after stopping these medications (the wash-out period is at least 4 weeks). In this case, the routine follow-up period for the 2nd visit (6 weeks after enrollment) will be prolonged assuming no violation of the study protocol has taken place.

All the subjects will be educated about common adverse events related to the eradication medications, and they will be asked to submit self-report questionnaires about adverse events related to the eradication medications at the 1st visit (2 weeks after enrollment, after ending the eradication regimen) and at the 2nd visit (6 weeks after enrollment, for UBT checking). The severity of the adverse events is defined as “mild” (transient and well tolerated), “moderate” (causing discomfort and partially interfering with daily activities), or “severe” (causing considerable interference with daily activities) and will be taught to the subjects for reporting as described above.^[20]^ Potential adverse events will be monitored by the research staff for 3 months after enrollment. Information about adverse events related to the eradication medications will also be gathered through interviews conducted by the doctors and independent staff at the 1st and 2nd visits. Compliance with the eradication regimen will be checked at the 1st visit by independent staff.

### 2.4 Randomization and blinding

Eligible patients will be approached during their routine gastroenterology unit visit by the research team for recruitment. A single independent staff member will prepare the randomization sequence, which will be accomplished using a block design and a block size of 4. Randomization of a block will be done by means of a random-number chart. This study is an open-label trial due to the difference in administration method for each arm. The modified quadruple therapy group will have amoxicillin twice a day, whereas the bismuth-containing quadruple therapy group will have tetracycline 4 times a day. Therefore, doctors and participants will be aware of the drugs that are prescribed. However, only the rate of eradication success, adverse events, and compliance with the therapies will be considered outcomes of this trial, and it is believed that an open-label design in itself will not influence the procedure or results of the trial.

### 2.5 Phenotypic and genotypic antibiotic resistance test

For the subjects who will prospectively undergo endoscopic examination with the rapid urease test or a histological examination to determine *H pylori* infection, 1 biopsy specimen from the gastric corpus or antrum will be cultured with an agar dilution test to assess phenotypic antibiotic resistance, and 1 specimen will be used to sequence the 23S rRNA gene in clarithromycin-resistant strains to assess genotypic antibiotic resistance. For the subjects who already underwent endoscopic examination or who were diagnosed with *H pylori* infection using the UBT, antibiotic resistance tests will be omitted.

The biopsy specimen will be cultured and maintained on brucella broth agar supplemented with 5% sheep blood and containing vancomycin (10 μg/mL), trimethoprim (5 μg/mL), amphotericin B (5 μg/mL), and polymyxin B (2.5 IU) under microaerophilic conditions (5% O_2_, 10% CO_2_, 85% N_2_) at 37°C for 3 to 7 days. All isolates will be stored in brucella broth supplemented with 15% glycerol at −70°C. A culture will be considered positive if 1 or more colonies are Gram-negative and positive for urease, oxidase, and catalase with a spiral/curved rod morphology. We will obtain 1 isolate per subject, and all of the stored isolates will be thawed and passaged for the agar dilution test.^[21]^

Thawed isolates will be inoculated onto Mueller-Hinton agar supplemented with 5% defibrinated sheep blood for 48 hours. Bacterial suspension adjusted to 1 x 10^7^ colony-forming units (equivalent to that of a No.2 McFarland opacity standard by spectrophotometry) will be inoculated directly onto an antibiotic-containing agar dilution plate at various concentrations. After 72 hours of microaerophilic incubation, the minimal inhibitory concentrations (MIC) of amoxicillin, clarithromycin, metronidazole, tetracycline, and levofloxacin will be determined by the 2-fold agar-dilution method. All 3 susceptibility tests will be performed for each sample. The breakpoint of MICs will be defined according to the criteria of the National Committee for Clinical Laboratory Standards (MIC value >0.5 μg/mL (amoxicillin), >1 μg/mL (clarithromycin), >8 μg/mL (metronidazole), >4 μg/mL (tetracycline), and >1 μg/mL (levofloxacin)), and ATCC 43504 (ATCC, Manassas, VA) will be used as the reference strain.^[22,23]^

*H pylori* genomic DNA will be extracted from biopsied tissue with the MagNA Pure 96 DNA and Viral NA Small Volume Kit (Roche Diagnostics, Forrenstrasse, Switzerland) according to the manufacturer's instructions. We will examine the nucleotide sequence of domain V in the 23S rRNA gene of *H pylori* by amplifying approximately the first 300 bp to detect mutations at positions 2141, 2142, 2143, 2144, and 2182. Nucleotide sequencing of the amplified DNA will be performed using an ABI 3730 DNA Analyzer (Applied Biosystems, Foster City, CA) with BigDye Terminator V3.1 according to the manufacturer's instructions. All end-point PCR reactions will be visualized with agarose gel electrophoresis, and all sequencing work will be performed by a commercial expert agency (Samkwang Medical Laboratories, Seoul, Korea).

### 2.6 Statistical analysis

The sample size was calculated by using an α-error <0.05 and a β-error <0.2 for a 2-tailed significance test. The calculated number of subjects required for the trial is 100 in each arm, which was determined by assuming an eradication rate of 96.2% from treatment with bismuth-containing quadruple therapy for 14 days as seen in a previous study in Korea (per-protocol (PP) analysis), an equivalence margin of 8%, and a dropout rate of 10%.^[24]^

**Figure 1:**
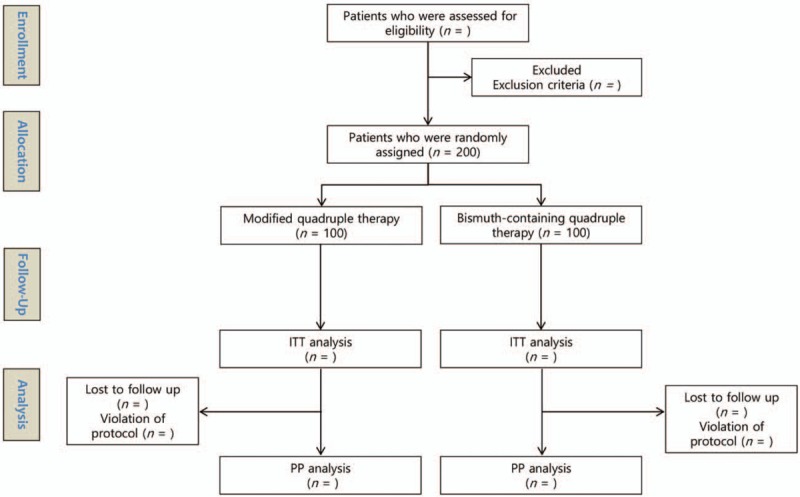
Flow chart of the study. ITT = intention-to-treat, n = number, PP = per-protocol.

For the intention-to-treat (ITT) analysis, all participants who take at least the 1st dose of prescribed medications will be included and assessed and for the modified ITT analysis, all participants who take at least the 1st dose of prescribed medications and who are in the posttreatment *H pylori* status will be included and assessed. For the PP analysis, only those who maintain and complete treatment with the prescribed medications without violating the regulations (violation is <80% medication compliance; poor adherence to medication is defined as taking less than 80% of what is prescribed) will be included and assessed (Fig. 1).

Continuous variables will be expressed as the mean ± standard deviation. Categorical variables will be expressed as numbers and percentages. First, we will compare the baseline characteristics of enrolled subjects (age, sex, smoking, alcohol use, reason for *H pylori* eradication) using Student *t* test and Fisher exact test for continuous and categorical variables, respectively. The Mann–Whitney test will be alternatively used if a continuous variable does not have a normal distribution. We will then perform ITT, modified ITT, and PP analyses to compare the eradication success rates, compliance with therapies, and adverse event rate between the 2 arms. Subgroup analyses will be performed according to the reason for eradication and the antibiotic resistance profile of the *H pylori* isolate. A *P* value <.05 (2-tailed) will be adopted as the threshold for statistical significance for all tests. All of the analyses will be performed using SPSS version 24.0. (SPSS Inc., Chicago, IL).

### 2.7 Data management

A common case report form (CRF) was produced by investigators and approved by the Ministry of Food and Drug Safety (MFDS) in Korea and the institutional review board (IRB) for human research of each hospital. Only designated members of the clinical trial staff will be allowed to record and correct data in the CRFs. Any personal identifiable variable will be recorded anonymously, and the common data form will be produced by the principal investigator at the end of enrollment. Any serious adverse events identified during the clinical trial will be reported immediately to the MFDS and the IRB for human research of each hospital, and confidential data can then be released and provided for the purpose of the safety of participants and for determining whether the clinical trial should be halted.

### 2.8 Ethics

The study protocol adheres to and will be conducted in accordance with the ethical guidelines established by the 1975 Declaration of Helsinki and the Food and Drug Administration regulations regarding good clinical practice and follows the SPIRIT guidelines for reporting protocol items for randomized controlled trials.^[25]^ We had received an approval by the MFDS in Korea and the IRB for human research at Hallym University Chuncheon Sacred Heart Hospital (2018–27) and Hallym University Sacred Heart Hospital (2018–06–008) before the study was initiated. This study was registered at ClinicalTrial.gov in June 2018 (Clinical trial registration number, NCT03665428). Informed consent to participate in the study will be obtained from each subject after providing an explanation of the potential benefits and harms with sufficient time for discussion, and the subjects who voluntarily want to participate in this study will be enrolled. All the participants will be educated by the doctors who prescribe the medication and by the research staff. They will be informed about the drug, administration time, possible adverse events and how to report adverse reactions. All potential serious adverse events suffered by subjects in the clinical trial will be managed by each hospital under the coverage of clinical trial insurance. Participants may withdraw from this trial for any reason without penalty; however, the reasons for withdrawal will be identified for reporting purposes.

## 3 Discussion

The clarithromycin-containing triple regimen for *H pylori* eradication is no longer acceptable due to a high clarithromycin resistance in Korea, and concomitant therapy or bismuth-containing quadruple therapy is recommended as an alternative regimen.^[8,9,12,13]^ However, Korea is classified as a region with high clarithromycin (>15%) and metronidazole resistance (>30%).^[9,16]^ Data on the trends over time have shown a constant acceptable eradication success rate for bismuth-containing quadruple therapy as a 2nd-line regimen;^[24,26,27]^ however, there is a paucity of data on its use as a 1st-line regimen in Korea.^[28]^ Moreover, there is no definite 2nd-line or 3rd-line rescue regimen for patients who do not respond to the above therapies.^[17]^ Fluoroquinolone- and rifabutin-containing regimens or a combination of bismuth with different antibiotics is the remaining empirical option.^[8,17]^ However, the antibiotic resistance rates in Korea of ciprofloxacin, levofloxacin, and moxifloxacin are reported to be up to 38.2%, 37.7%, and 34.6%, respectively, which are higher than those previously reported.^[15,17,29]^ Cross-resistance between rifabutin and rifampin is a serious concern in tuberculosis prevalent countries such as Korea due to concerns that overuse may increase the prevalence of rifabutin-resistant mycobacteria in the community.^[17]^ Antibiotic susceptibility guided treatment is beginning to receive more attention; however, it is not widely available at all institutions.^[17]^

In a meta-analysis of Korean studies, concomitant therapy showed a higher eradication rate than sequential therapy.^[30]^ Though there has been no published data on its trends over time, the continuous eradication success of concomitant therapy can be demonstrated through the enrolled studies in a recent systematic review of Korean studies.^[31]^ However, concomitant therapy is not recommended in regions with high metronidazole resistance, and the resistance rate of metronidazole has been reported to be as high as 66% in Korea.^[9,16]^ Moreover, dual clarithromycin and metronidazole resistance undermines the efficacy of concomitant therapy.^[18]^

A recent study in Korea has shown that modified quadruple therapy has rates of eradication success and adverse events comparable to those of concomitant therapy.^[19]^ Characterizing the regional antibiotic resistance profile is important for *H pylori* eradication because antibiotic resistance is the chief cause of treatment failure. However, the study above did not explore antibiotic resistance profiles, making it difficult to interpret its results. A previous study has also shown that modified quadruple therapy is comparable to bismuth-containing quadruple therapy as a rescue regimen for patients with at least 2 consecutive treatment failures.^[20]^ Additionally, modified quadruple therapy has been shown to have high eradication success even in subjects with amoxicillin, metronidazole, or clarithromycin resistance, and this therapy has fewer adverse events and higher compliance than does bismuth-containing quadruple therapy.^[20]^

This study will compare the efficacy and safety of modified quadruple therapy and bismuth-containing quadruple therapy as well as presenting the phenotypic and genotypic antibiotic resistance profile of *H pylori*. The strength of this study resides in providing evidence regarding the efficacy and safety of using modified quadruple therapy as a 1st-line regimen in different regions of Korea compared to previous reports (a previous study was conducted in Seoul as a single center study; however, we will recruit patients in Gangwon and Gyeonggi provinces) and additionally, in providing evidence on the use of bismuth-containing quadruple therapy as a 1st-line treatment, which has been scarce as a 1st-line treatment in Korea. Moreover, with the comparative results of the antibiotic-susceptibility gold standard ‘agar dilution’ method vs. DNA sequencing of the clarithromycin resistance point mutations in the 23S rRNA of *H pylori*, it will be possible to interpret the outcome of this study in light of the antibiotic resistance profiles of *H pylori* including the unknown clinical significance of point mutations, such as G2141A, A2144T, or T2182C.

## Author contributions

**Conceptualization:** Chang Seok Bang, Woon Geon Shin.

**Data curation:** Hyun Lim, Chang Seok Bang, Woon Geon Shin, Jae Ho Choi, Jae Seung Soh, Ho Suk Kang, Young Joo Yang, Ji Taek Hong, Suk Pyo Shin, Ki Tae Suk, Jae Jun Lee, Gwang Ho Baik, Dong Joon Kim.

**Formal analysis:** Hyun Lim, Chang Seok Bang, Woon Geon Shin.

**Funding acquisition:** Chang Seok Bang.

**Investigation:** Hyun Lim, Chang Seok Bang, Woon Geon Shin, Jae Ho Choi, Jae Seung Soh, Ho Suk Kang, Young Joo Yang, Ji Taek Hong, Suk Pyo Shin, Ki Tae Suk, Jae Jun Lee, Gwang Ho Baik, Dong Joon Kim.

**Methodology:** Chang Seok Bang, Woon Geon Shin.

**Project administration:** Chang Seok Bang.

**Resources:** Chang Seok Bang, Woon Geon Shin.

**Software:** Chang Seok Bang.

**Supervision:** Chang Seok Bang.

**Validation:** Chang Seok Bang.

**Visualization:** Chang Seok Bang, Woon Geon Shin.

**Writing – original draft:** Hyun Lim, Chang Seok Bang.

**Writing – review & editing:** Chang Seok Bang, Woon Geon Shin.

Chang Seok Bang orcid: 0000-0003-4908-5431

## References

[1] Schistosomes, liver flukes and Helicobacter pylori. IARC Working Group on the Evaluation of Carcinogenic Risks to Humans. Lyon, 7-14 June 1994. IARC Monogr Eval Carcinog Risks Hum. 1994;61:1–241. PMC7681621 7715068

[2] Kwak HW Choi IJ Cho SJ Characteristics of gastric cancer according to *Helicobacter pylori* infection status. J Gastroenterol Hepatol 2014;29:1671–7. 24730518 10.1111/jgh.12605

[3] Correa P A human model of gastric carcinogenesis. Cancer Res 1988;48:3554–60. 3288329

[4] Choi IJ Kook MC Kim YI *Helicobacter pylori* therapy for the prevention of metachronous gastric cancer. N Engl J Med 2018;378:1085–95. 29562147 10.1056/NEJMoa1708423

[5] Lee YC Chiang TH Chou CK Association between *Helicobacter pylori* eradication and gastric cancer incidence: a systematic review and meta-analysis. Gastroenterology 2016;150:1113–24. 26836587 10.1053/j.gastro.2016.01.028

[6] Ford AC Forman D Hunt R Helicobacter pylori eradication for the prevention of gastric neoplasia. Cochrane Database Syst Rev 2015;7:CD005583. 10.1002/14651858.CD005583.pub2 PMC7263416 26198377

[7] Bang CS Baik GH Shin IS *Helicobacter pylori* eradication for prevention of metachronous recurrence after endoscopic resection of early gastric cancer. J Korean Med Sci 2015;30:749–56. 26028928 10.3346/jkms.2015.30.6.749 PMC4444476

[8] Malfertheiner P Megraud F O’Morain CA Management of *Helicobacter pylori* infection-the maastricht v/florence consensus report. Gut 2017;66:6–30. 27707777 10.1136/gutjnl-2016-312288

[9] Suzuki H Mori H World trends for *H. pylori* eradication therapy and gastric cancer prevention strategy by *H. pylori* test-and-treat. J Gastroenterol 2018;53:354–61. 29138921 10.1007/s00535-017-1407-1 PMC5847180

[10] Lee JH Choi KD Jung HY Seroprevalence of *Helicobacter pylori* in Korea: a multicenter, nationwide study conducted in 2015 and 2016. Helicobacter 2018;23:e12463. 29345022 10.1111/hel.12463 PMC5900911

[11] Globocan Cancer Observatory. Available at: http://gco.iarc.fr Accessed on Sep 13, 2018.

[12] Kim SG Jung HK Lee HL Guidelines for the diagnosis and treatment of *Helicobacter pylori* infection in Korea, 2013 revised edition. J Gastroenterol Hepatol 2014;29:1371–86. 24758240 10.1111/jgh.12607

[13] Gong EJ Yun SC Jung HY Meta-analysis of first-line triple therapy for *Helicobacter pylori* eradication in Korea: is it time to change. J Korean Med Sci 2014;29:704–13. 24851029 10.3346/jkms.2014.29.5.704 PMC4024949

[14] Kim BJ Kim HS Song HJ Online registry for nationwide database of current trend of helicobacter pylori eradication in korea: interim analysis. J Korean Med Sci 2016;31:1246–53. 27478335 10.3346/jkms.2016.31.8.1246 PMC4951554

[15] Lee JW Kim N Kim JM Prevalence of primary and secondary antimicrobial resistance of Helicobacter pylori in Korea from 2003 through 2012. Helicobacter 2013;18:206–14. 23241101 10.1111/hel.12031

[16] Kim N Kim JM Kim CH Institutional difference of antibiotic resistance of *Helicobacter pylori* strains in Korea. J Clin Gastroenterol 2006;40:683–7. 16940878 10.1097/00004836-200609000-00004

[17] Choi JH Yang YJ Bang CS Current status of the third-line *Helicobacter pylori* eradication. Gastroenterol Res Pract 2018;2018:6523653. 29853863 10.1155/2018/6523653 PMC5954858

[18] Huang CC Tsai KW Tsai TJ Update on the first-line treatment for Helicobacter pylori infection—a continuing challenge from an old enemy. Biomark Res 2017;5:23. 28702193 10.1186/s40364-017-0103-x PMC5505131

[19] Choe JW Jung SW Kim SY Comparative study of *Helicobacter pylori* eradication rates of concomitant therapy vs modified quadruple therapy comprising proton-pump inhibitor, bismuth, amoxicillin, and metronidazole in Korea. Helicobacter 2018;23:e12466. 29369454 10.1111/hel.12466

[20] Chen Q Zhang W Fu Q Rescue therapy for *Helicobacter pylori* eradication: a randomized non-inferiority trial of amoxicillin or tetracycline in bismuth quadruple therapy. Am J Gastroenterol 2016;111:1736–42. 27670603 10.1038/ajg.2016.443

[21] Kim JM Kim JS Jung HC Virulence factors of *Helicobacter pylori* in Korean isolates do not influence proinflammatory cytokine gene expression and apoptosis in human gastric epithelial cells, nor do these factors influence the clinical outcome. J Gastroenterol 2000;35:898–906. 11573725 10.1007/s005350070003

[22] National Committee for Clinical Laboratory Standards. Performance standards for antimicrobial susceptibility testing; seventeenth informational supplement. Available at: http://www.microbiolab-bg.com/CLSI.pdf Accessed on Sep 13, 2018.

[23] Kim JM Kim JS Kim N Distribution of fluoroquinolone MICs in Helicobacter pylori strains from Korean patients. J Antimicrob Chemother 2005;56:965–7. 16159928 10.1093/jac/dki334

[24] Chung JW Lee JH Jung HY Second-line Helicobacter pylori eradication: a randomized comparison of 1-week or 2-week bismuth-containing quadruple therapy. Helicobacter 2011;16:289–94. 21762268 10.1111/j.1523-5378.2011.00844.x

[25] Chan AW Tetzlaff JM Gotzsche PC SPIRIT 2013 explanation and elaboration: guidance for protocols of clinical trials. BMJ 2013;346:e7586. 23303884 10.1136/bmj.e7586 PMC3541470

[26] Shin WG Lee SW Baik GH Eradication rates of *Helicobacter pylori* in Korea over the past 10 years and correlation of the amount of antibiotics use: nationwide survey. Helicobacter 2016;21:266–78. 26470999 10.1111/hel.12279

[27] Kim SE Park MI Park SJ Second-line bismuth-containing quadruple therapy for Helicobacter pylori eradication and impact of diabetes. World J Gastroenterol 2017;23:1059–66. 28246480 10.3748/wjg.v23.i6.1059 PMC5311095

[28] Jang HJ Choi MH Kim YS Effectiveness of triple therapy and quadruple therapy for *Helicobacter pylori* eradication. Korean J Gastroenterol 2005;46:368–72. 16301850

[29] Lee JY Kim N Kim MS Factors affecting first-line triple therapy of *Helicobacter pylori* including CYP2C19 genotype and antibiotic resistance. Dig Dis Sci 2014;59:1235–43. 24599773 10.1007/s10620-014-3093-7

[30] Bae HJ Kim JS Kim BW Concomitant or sequential therapy as the first-line therapy for eradication of *Helicobacter pylori* infection in Korea: a systematic review and meta-analysis. Korean J Gastroenterol 2018;71:31–7. 29361811 10.4166/kjg.2018.71.1.31 PMC12285810

[31] Jung YS Park CH Park JH Efficacy of *Helicobacter pylori* eradication therapies in Korea: a systematic review and network meta-analysis. Helicobacter 2017;22. 10.1111/hel.12389 28425141

